# Supplementary material for: A systematic review and meta-analysis of vineyard techniques used to delay ripening
Source: Hortic Res. 2022 May 17;9:uhac118. doi: 10.1093/hr/uhac118 (PMC9343921; doi:10.1093/hr/uhac118)
Supplement: Web_Material_uhac118 [file web_material_uhac118.docx]

A systematic review and meta-analysis of vineyard techniques used to delay ripening

Pietro Previtali^1,2*^, Filippo Giorgini^3^, Randall S. Mullen^4^, Nick K. Dookozlian^2,5^, Kerry L. Wilkinson^1,2^, Christopher M. Ford^1,2^

^1^ Department of Wine Science and Waite Research Institute, The University of Adelaide, PMB1 Glen Osmond, SA, 5064, Australia

^2^ Australian Research Council Training Centre for Innovative Wine Production, PMB1 Glen Osmond, SA, 5064, Australia

^3^ Department of Economy, Management and Statistics, University of Milano-Bicocca, I-20125 Milano, Italy

^4^ Research and Development Statistics, E. & J. Gallo Winery, Modesto, CA 95354, USA

^5^ Department of Winegrowing Research, E. & J. Gallo Winery, Modesto, CA 95354, USA

*** Correspondence:**

P. Previtali ([pietro.previtali@adelaide.edu.au](mailto:pietro.previtali@adelaide.edu.au))

**Short title:** Delayed ripening: a meta-analysis

**Email addresses:**

FG: [f.giorgini@campus.unimib.it](mailto:f.giorgini@campus.unimib.it), RM: [randall.mullen@ejgallo.com](mailto:randall.mullen@ejgallo.com),ND: [nick.dokoozlian@ejgallo.com](mailto:nick.dokoozlian@ejgallo.com), KW: [kerry.wilkinson@adelaide.edu.au](mailto:kerry.wilkinson@adelaide.edu.au), CF: [christopher.ford@adelaide.edu.au](mailto:christopher.ford@adelaide.edu.au)

Abstract

Several vineyard techniques have been proposed to delay grape maturity in light of the advanced maturation driven by increasingly frequent water and heat stress events that are detrimental to grape quality. These studies differ in terms of their experimental conditions, and in the present work we have attempted to summarize previous observations in a quantitative, data-driven systematic review. A meta-analysis of quantitative data gathered across 43 relevant studies revealed the overall significance of the proposed treatments and evaluated the impact of different experimental conditions on the outcome of antitranspirants, delayed pruning and late source limitation. Antitranspirants were most effective when applied twice and closer to veraison, while di-1-*p*-menthene increased the ripening delay by about 1 °Brix compared to kaolin. Larger ripening delays were achieved with delayed pruning of low-yielding vines or by pruning at later stages of apical bud development. Late defoliation or shoot trimming delayed ripening in high-yielding vines and represent suitable solutions for late-harvested varieties, but became ineffective where the treatment decreased yield. This quantitative meta-analysis of 242 primary observations uncovers factors affecting the efficacy of vineyard practices to delay ripening, which should be carefully considered by grape growers attempting to achieve this outcome.

Keywords: antitranspirants, berry quality, delayed pruning, grapevine, late source limitation, vineyard management, *Vitis vinifera*

# Introduction

Horticultural crops are extremely sensitive to environmental conditions that can affect production both quantitatively (i.e. yields) and qualitatively (i.e. quality traits). In wine grapes, the relationship between yield and quality is of primary importance. Research conducted over past decades has shown that many specialized metabolites in grapes including phenolic and aroma compounds carry through the fermentation process to define wine sensory profiles^1^.

In a scenario of changing climate conditions, grape cultivation has been affected at multiple levels, and changes in grapevine phenology, physiology and grape ripening have been reported elsewhere^2–5^. Accelerated grape maturation led by faster sugar accumulation is of primary concern for the wine growing sector^3^. Under these conditions a decoupling between sugars (technological maturity) and secondary metabolites of grapes (phenolic and aromatic maturity) has been observed^6,7^. This signifies that grapes harvested at the desired technological maturity have reached only a sub-optimal phenolic/aromatic maturity or, conversely, grapes picked at the targeted aromatic/phenolic maturity display supra-optimal sugar concentrations. Vintage compression is another detrimental effect of accelerated ripening, which imposes serious threats to wineries with regards to fruit intake, management and storage capacity^8^. In response to these concerning trends, efforts have been made to investigate vineyard operations to delay grape maturity. A review published in 2014 reported early findings on the topic and suggested changes in vineyard management to counteract climate change-related detriments to grape quality^9^ and a recent review has incorporated newer studies published thereafter^10^. With an increasing frequency of reports focusing on delayed ripening, sets of data have become available in which numerous varieties, regions, environmental and experimental conditions were tested. However, review studies on the topic are highly qualitative and a quantitative summary of previous data is not available.

Meta-analysis (MA) utilizes statistical methods to compare outcomes of a specified treatment [referred to as “effect size” (ES)] across multiple similar studies^11^. This approach is entirely quantitative and in combination with the systematic review procedure allows fully data-driven interpretations of the efficacy of the treatment investigated. Although MA has been applied mostly to medical or psychological trials, recent publications in agronomy have adopted it, including a limited number of papers in grapes and wine^12–15^. As a consequence of the use of this tool gaining momentum, guidelines for its correct use and the interpretation of MA data in agronomy have also been made available^16,17^.

The present study is a systematic review of vineyard techniques to delay ripening. Among the effects of climate change on viticulture, here we tackled the accelerated sugar accumulation by gathering Total Soluble Solids (TSS) values from a wide range of studies. Sets of data were then rigorously analyzed using MA to test significant differences in TSS between treated and control grape material. Following a general analysis across all strategies, the role of different experimental conditions at the genotypic, environmental and viticultural levels was dissected within individual strategies. This aimed to explain the variation of treatment effects across different studies and identify conditions under which the studied strategies were more effective.

# Material and methods

## Research question and selected response variable

The aim of this MA was to investigate whether previous data provide enough evidence that vineyard operations can effectively cause a ripening delay. ES values (i.e. ripening delays) were characterized as differences in the sugar concentration of treated and control grapes on the same day. This allowed us to incorporate a quantitative measure of the ripening delay in addition to the presence/absence of the delay, represented by positive and negative ES values respectively. The response variable of interest ES was calculated as described in **Equation 1**.

$ES= {TSS}_{C}-{TSS}_{T}$ (1)

where ${TSS}_{C}$and ${TSS}_{T}$ are the TSS of the control and treated group respectively, expressed using the °Brix scale. Data reported using other scales (e.g. °Baume) were converted using tabulated values. ES values were not standardized using transformations, such as Cohen’s *d* and Hedges’ *g*, as was done in other meta-analyses^18^. This was done to avoid problems arising when applying such transformations^19^ and because untransformed TSS values were considered more meaningful from a viticultural viewpoint, as decreases or increases in TSS on the same day can be readily translated into a quantitative measure of ripening advancements or delays. Standard errors (SE) of the ES were calculated according to **Equation 2** as follows:

${SE}_{ES}= \sqrt{{{SE}_{C}}^{2}+{{SE}_{T}}^{2}}$ (2)

where${SE}_{C}$and${SE}_{T}$were the standard errors of the control and treated group respectively. This formula normally includes a correction term related to the correlation between the two groups. Herein it was assumed that there was independence of the treated and control groups and therefore the correction was omitted. In the absence of different specifications, the sizes of the two groups were considered to be equal. When only one SE was available, it was assumed that variances of the two groups were equal.

## Literature search

The literature search was conducted by the first author in the following databases listed in order of consultation: Web of Science Core Collection, PubMed and Google Scholar. In Web of Science (search results: 26), the search was conducted using the following key: TI = (grape* OR *Vitis vinifera* OR berries) AND TI = (delay* OR late) AND TI = (ripening OR sugar OR maturity) NOT TI = (transcript*). In PubMed (search results: 6): (((grape*[Title] OR *Vitis vinifera*[Title]) AND (delay*[Title] OR late[Title])) AND (ripening[Title] OR sugar[Title] OR maturity[Title])) NOT (transcr*[Title]). At this stage, duplicates were removed and irrelevant studies were discarded based on title and abstract information. Papers were classified in macro-categories, each one corresponding to a treatment applied to delay ripening: auxin treatment, antitranspirants, delayed pruning, late defoliation, late season irrigation and late trimming. Using these categories as additional keywords, the search was then extended to Google Scholar and proceedings of the main conferences in the field (search results: 4): GiESCO, International Symposium of Grapevine Physiology and Biotechnology, CO.NA.VI (through BIO Web of Conferences journal). Based on the first author’s knowledge of the active research groups in the field of interest, researchers’ websites and ResearchGate pages were also consulted. Steps in data curation were tracked following the ‘Preferred reporting items for meta-analysis’ (PRISMA) statement^20^. To interpret data of other authors with the least subjectivity and maximize data-driven outputs, studies were de-identified using ID numbers.

## Inclusion and exclusion criteria

Inclusion and exclusion criteria for the MA were initially applied by the first author and then repeated by the second author and are listed here below in the same order as they were used for study selection.

1. **Publication quality requirements:** we included published original articles, pre-print articles, books, industry reports, technical reports, dissertations, theses and conference proceedings written in English. Articles and proceedings were only included if published in journals and conferences related to the field of agriculture and food, preferentially viticulture and oenology.
2. **Publication year:** only material published between 2000 and 2020 was considered eligible.
3. **Availability of data of interest:** only studies that reported TSS measured in treated and control grapes (means ± SE or SD) on the same day, preferentially when control reached the targeted commercial maturity, were selected. Numeric values of means and SE per treatment were preferred but very often were replaced by graphics of TSS accumulation over time (i.e. ripening kinetics) in the original articles. In many instances tabulated data were provided but SE values were missing as per the common use of compact letter display to indicate significant differences. In case of missing data, a first attempt consisted in retrieving means and SE values from graphics using the software ImageJ I.x with the ‘*Figure Calibration’* plugin^21^. This was done only if the type of dispersion measure utilized (SD vs SE) and number of replicates (n) were specified in figure captions or methodology sections. The equality between graph-derived and actual data was tested on a set of TSS (114) and SE (105) values available in both numerical and graphical formats using the Passing-Bablock regression (the full analysis is reported in the **Supporting Information**, section 3.3.3)^22^. When it was not possible to retrieve data from graphics, corresponding authors were contacted to request TSS data. TSS datasets of twenty-three papers were requested, with eleven replies and datasets provided for seven studies.
4. **Validity of design and statistical analysis:** requirements for inclusion were that the study was of a randomized design, conducted in the field (no greenhouse or potted vines), with 2 or more replicates per treatment. Only data analyzed with relevant statistical tests were included, as these details were necessary to calculate *p*-values for the ES values.
5. **Multifactorial designs and multiple vintages:** in studies conducted over multiple vintages, individual ES values per vintage were included separately. Similarly, in multifactorial studies (e.g. combining crop load manipulation and differential irrigation), we included separate ES values of the delaying treatment within each level of the second factor investigated.

## Explanatory variables

Categorical and numerical variables annotated from original papers were used to summarize the geographical, environmental and experimental conditions for each of the ES values. Growing degree days (GDDs) were used to approximate temperature trends at the experimental sites for each vintage, as this parameter was widely reported in the original studies. To standardize our methodology, GDDs were calculated in the period between 1 Apr-31 Oct for the northern hemisphere and 1 Oct-30 Apr for the southern hemisphere and using a base temperature of 10 °C^23^. In case of missing GDDs or differences in calculation boundaries or base temperatures, GDDs were extrapolated from weather databases. Local or regional databases were consulted (**Supporting Information**, Section 2.1) as well as the Global Surface Summary of the Day (GSOD) weather station network (United States National Oceanic and Atmospheric Administration National Center for Environmental Information 2020). GSOD data of temperature and rainfall were obtained using the *‘GSODR*’ package in R^24^. Using geographical coordinates of the experimental sites, GSOD stations were identified as close to the vineyard as possible and daily precipitation and temperature data downloaded.

## Exploratory analysis

The dataset for qualitative synthesis was submitted to exploratory analysis (EA), comprising three sections. The traditional EA investigated the distribution of ES values and studies according to publication and experimental factors. The frequency and association of keywords and title words was also explored. In the authority ranking section, the number of citations and the design of the experiment were investigated to verify the validity of the studies. Functional EA explored different statistical methods applied in the original papers and the distribution of ES and SE values based on the way they were obtained from original studies.

## Meta-analysis

### *P*-curve

Publication bias among all papers was investigated using the p-curve method^25^. This methodology assumes that *p*-values are uniformly distributed, associated with the hypothesis of interest and statistically independent. Accordingly, only *p*-values meeting these assumptions were included. For papers with multiple *p*-values, the median of all *p*-values below 0.05 was utilized. The *p*-curve was produced using the *p*-curve app version 4.06 available online ([www.p-curve.com](http://www.p-curve.com)).

### PET-PEESE

Precision effect test (PET) was performed on the full dataset following guidelines previously reported^26^. Briefly, ES values were regressed on their SE values using a weighted least squared regression in which weights were represented by the inverse variance of the ES values. The only modification was the addition of treatment categories as a random effect on the intercept. Following authors’ recommendations, the true estimate of the ES was corrected using the precision effect estimate with standard error (PEESE) technique^26,27^.

### Preliminary analysis of individual treatment categories

It was assumed that ES values were derived from different populations, therefore random effects models within each treatment category were fitted to estimate the standardized mean difference (SMD) of the overall ES. The Sidik-Jonkman estimator of the variance of true effect sizes (τ^2^) was employed^28^. Forest plots were used to summarize SMD estimates and confidence intervals (CIs) for single studies and as a preliminary analysis of the overall effect and heterogeneity (*I*^2^) across the studies. Funnel plots were produced to check publication bias within each treatment category as reported in ^11^.

### Meta-regression

Three treatment categories were selected that had a sufficient number ES values (n > 35), namely antitranspirants (AT), delayed pruning (DP) and late source limitation (LSL). For each of these treatments, a meta-regression was used to understand the effect of experimental conditions of interest on treatment outcomes. Variables of interest were carefully selected for each model and their selection was driven by knowledge of key factors from a viticultural perspective as well as data availability. GDDs, rainfall and their interaction (GDDs × rainfall) were included in models for all three treatments to explore the impact of climatic conditions. As GDDs and rainfall data were 1000-fold and 100-fold larger than ES values, these terms were both divided by 100 to fit the model and then back-transformed in model outputs. Another common term across the models was TSS_Control_, which was the TSS maturity (in °Brix) of control grapes on the same date at which ES was calculated. Introducing this term allowed exploration of possible changes to the ES caused by harvesting at variable targeted TSS levels, with implications for industry as varying ranges of TSS are targeted for different varieties and desired wines styles. In addition to these common fixed effects, treatment-specific models are outlined below along with explanation of the corresponding variables of interest.

#### Antitranspirants

The statistical linear mixed model fitted to explain ripening delays achieved by application of AT, fitted to 102 observations (full dataset available as “*DB1_Antitranspirants*” at the link provided in the **Data Availability Statement**), is described in **Equation 3** as follows:

| ${ES}_{AT}=\left( \beta_{0}+\lambda_{site \times vintage} \right)+\beta_{GDDs}+\beta_{Rainfall}+\beta_{GDD \times Rainfall}+\beta_{{TSS}_{Control}}+\beta_{Ingredient}+\beta_{Timing}+\beta_{Second}+\varepsilon$ | (3) |
| --- | --- |

where $\beta_{0}$is the intercept, the following $\beta$ values are regression coefficients for each fixed term, $\lambda$ represents the contribution of random effects and $\varepsilon$ the residual random variance. The interaction of *site* × *vintage* was fitted as random term on the intercept ${(\lambda}_{site \times vintage})$. Models weighted by SE_ES_ or the number of replicates (N) were tested but did not increase the predictive power (**Supporting Information**, Section 4.1.3). *Ingredient* was the active component of the product sprayed (i.e. kaolin *vs* di-1-*p*-menthene). *Timing* reflected the phenological stage at which antitranspirants were sprayed, related to the BCCH stage according to ^29^ and expressed in ordered categorical codes. *Second* was created as a dummy variable indicating whether the treatment was repeated at a second stage (level 1) or not (level 0).

#### Delayed pruning

The linear mixed model utilized to explore factors affecting the ripening delay achieved with DP, fitted to 45 observations (full dataset available as “*DB2_Delayed pruning*” at the link provided in the **Data Availability Statement**), is described in **Equation 4** as follows:

| ${ES}_{DP}=\left( \beta_{0}+\lambda_{vintage \times variety} \right)+\beta_{GDDs}+\beta_{Rainfall}+\beta_{GDD \times Rainfall}+\beta_{Buds/vine}+\beta_{{TSS}_{C}}+\beta_{Pre-pruning}+\beta_{Pruning stage}+\beta_{{Yield}_{Control}}+\beta_{{ES}_{YIELD}}+\varepsilon$ | (4) |
| --- | --- |

where $\beta_{0}$is the intercept, the following $\beta$ values are regression coefficients for each fixed term, $\lambda$ represents the contribution of random effects and $\varepsilon$ the residual random variance. The *vintage*×*variety* interaction was fitted as a random term on the intercept ($\lambda_{vintage \times variety}$). *Buds/vine* was a general indicator of vine size based on targeted bud counts. *Pre-pruning* was a dichotomous variable (Y/N) stating whether vines were pre-pruned and hand-finished or only pruned once. *Pruning stage* indicated the stages at which vines were pruned, fitted as categorical variable expressed using ordered stages of the BBCH scale^29^. As delayed pruning exploits the advanced development of apical buds versus basal buds, it is worth specifying here that phenological stages recorded in this variable referred to apical buds as in the original papers. Two variables referring to vine yield were added to the model, namely *ES_Yield_* and *Yield_Control_*. ES_Yield_ was the effect size calculated for yield, calculated as difference in yields between the control and the treated group, in the same order as they are listed. For standardization purposes, yields were converted to reflect kg/m of cordon using intra-row spacings and planting density as needed when yield data were reported in t/ha or kg/vine in original articles. The effect size is a quantitative measure of the magnitude of the experimental effect and, as such, does not account for absolute values in the control or treated groups. Therefore, *Yield_Control_* was fitted as an additional variable reporting absolute yields of control vines in each experimental trial, again expressed as kg/m of cordon.

#### Late source limitation

The linear mixed model utilized to explore factors affecting the ripening delay achieved by LSL, fitted to 56 observations (full dataset available as “*DB3_Late source limitation*” at the link provided in the **Data Availability Statement**), is described in **Equation 5** as follows:

| ${ES}_{LSL}=\left( \beta_{0}+\lambda_{variety} \right)+\beta_{GDDs}+\beta_{Rainfall}+\beta_{GDD \times Rainfall}+\beta_{Treatment type}+\beta_{Treatment timing}+\beta_{{TSS}_{Control}}+\beta_{{ES}_{LAFW}}+\beta_{{LAFW}_{Control}}+\beta_{{ES}_{YIELD}}+\beta_{{Yield}_{Control}}+\varepsilon$ | (5) |
| --- | --- |

where $\beta_{0}$ is the intercept, the following $\beta$ values are regression coefficients for each fixed term, $\lambda$ represents the contribution of random effects and $\varepsilon$ the residual random variance. In the model for late source limitation, *variety* was fitted as a random term on the intercept ($\lambda_{variety}$). *Treatment type* was a dichotomous variable specifying whether late apical defoliation or late shoot trimming was used. *Treatment timing* was the stage at which treatments were applied, recorded using numerical TSS values (in °Brix). A TSS level of 9 °Brix was used as a surrogate for veraison, according to earlier observations^30,31^. Yield and leaf area-to-fruit weight (LAFW) ratios were incorporated in the model. Similarly to the DP model, two parameters were fitted for each of these two physiological parameters, including their ES values (*ES_LAFW_* and *ES_Yield_*) and absolute levels in the control vines (*LAFW_Control_* and *Yield_Control_*).

## Statistical analysis

Data collation from single studies was done in Microsoft Excel (2020). The original and treatment-specific datasets are available at the link provided in the **Data Availability Statement** section. Statistical analysis was performed using R (R Foundation for Statistical Computing, Vienna, Austria) version 4.0.5 in RStudio (RStudio Inc., Boston, MA, USA) using a range of available packages and custom-made code. EA plots were produced using ‘*ggplot2*’, ‘*plotly*’ and ‘*leaflet’*^32–34^. Functions of the packages ‘*meta*’ and ‘*metafor*’ were utilized for MA of the data^35,36^. Linear mixed models were fitted using the ‘*lmerTest*’ package and results analyzed with various functions of ‘*lattice*’ and ‘*lme4*’^37–39^. Predictions and CIs for fixed and random terms were computed using the ‘*ggeffects*’ package^40^.

# Results

## Compilation of databases for qualitative and quantitative analysis

Steps of data curation are reported in [**Figure 1**](#Figure1) using the PRISMA statement layout^20^. The dataset for qualitative analysis, composed of 51 studies and 297 ES values, was submitted to EA. The full EA is reported in the **Supporting Information** (Section 3). The distributions of studies (n) and ES values (n_ES_) per treatment category in the dataset for qualitative synthesis were as follows: antitranspirants (n = 12, n_ES_ = 109); auxin treatment (n = 3, n_ES_ = 3); delayed pruning (n = 10, n_ES_ = 64); late defoliation (n = 7, n_ES_ = 32); late season irrigation (n = 4, n_ES_ = 12); late trimming (n = 8, n_ES_ = 38); peduncle girdling (n = 2, n_ES_ = 4).

In the database for quantitative synthesis (i.e. MA), all ES values for which SE were not reported were discarded, causing a further decrease in the number of studies and ES values (n = 43, n_ES_ = 242, [**Figure 1**](#Figure1)). The frequencies of studies and ES values in the MA dataset by treatment are discussed below.

## Publication bias and evidential value in the full dataset

The *p*-curve and PET-PEESE methodologies were first applied to the full set of quantitative data (n_ES_ = 242) for a preliminary investigation of the efficiency of vineyard practices to delay ripening.

The *p*-curve of studies on delayed ripening is reported in [**Figure 2A**](#Figure2) (blue line) and compared to a null effect curve (red dotted line), in which *p*-values are uniform, and a curve suggesting an adequate evidential value (green dotted line) as reported by the authors of this technique^25^. The distribution of significant *p*-values from the collection of studies of interest was significantly different compared to the baseline and displayed a marked right-skewed distribution. As reported in [**Section 2.6.1**](#_P-curve), our approach was to utilize the median *p*-value if multiple *p*-values were reported within a given study. Even adopting a more conservative approach, in which the largest *p*-value per study was selected instead of the median, the statistical conclusion of the *p*-curve test was unchanged (data not shown).

In the PET-PEESE model, different treatment categories were fitted as random terms on the intercept, which was significant at *p* < 0.001. Normality of the model residuals is shown in [**Figure 2B**](#Figure2). Results of the PET model showed a significant (*p* = 0.024) estimate of the intercept (1.52 °Brix; CI: 1.47 - 1.57). The corrected estimate of ripening delay obtained using PEESE was 1.28 °Brix (CI: 1.23 - 1.33). Different fits by treatments are shown in [**Figure 2C**](#Figure2), while [**Figure 2D**](#Figure2) shows the random effect of different treatments as deviation from the overall model intercept (represented by the red dotted line). Random effects were all positive and some displayed large CIs due to limited observations, namely auxin treatment (3.90 °Brix, n = 3), early trimming (0.36 °Brix, n = 8), late season irrigation (0.28 °Brix, n = 4) and peduncle girdling (1.62 °Brix, n = 3). The average ES values returned by PET-PEESE for the three treatments of interest were 1.22 (antitranspirants), 0.58 (delayed pruning), 0.98 (late defoliation) and 1.29 °Brix (late trimming).

## Meta-regression

Further investigation within individual treatments of interest (AT, DP, LSL) was performed using MA techniques applying the same workflow of analysis, which is briefly explained in the **Supporting Information** (Section 4) to aid interpretation of statistical outputs.

### Antitranspirants

The dataset of ATs summarized through MA consisted of 105 ES values retrieved from 12 studies. Experimental trials on ATs were conducted in Australia, China, Europe and the United States on 13 different grape varieties, including 5 white and 8 red cultivars (**Supporting Information**, Sections 3.1.9 to 3.1.11). Pooling together 95 ES values, the forest plot ([**Figure 3A**](#Figure3)) returned an average ES of 0.74 °Brix (CI: 0.54 – 0.94). ES values were symmetrically distributed around the CI of the estimated ES in the funnel plot ([**Figure 3B**](#Figure3)) and mostly within the 95 % confidence band. Heterogeneity among observations was high (*I^2^* = 0.95), reflecting large variation in experimental conditions which was further investigated using meta-regression.

The residuals of the AT meta-regression model were independent ([**Figure 3C**](#Figure3)) and normally distributed ([**Figure 3D**](#Figure3)), thus assumptions for the use of linear mixed models were verified. The final model for ATs is reported in [**Table 1**](#Table1). Models with different random effects on the intercept were compared (**Supporting Information**, Section 5.1.2) and the model with site × vintage as a random term was selected. Because vintage effects can be different depending on the site considered, the interaction between site and vintage was preferred to fitting the two random terms separately. Weighted models did not improve the predictive performance, thus the final model did not include weights (**Supporting Information**, Section 5.1.3). The effects of environmental factors associated with seasonal temperature (GDDs) and rainfall or their interaction were not significant. Among the experimental factors, TSS_Control_ was not significant, while active compounds and the timing and number of applications were found to have a significant effect on the ES. The seasonal rainfall was significant at *p* = 0.078 in the final model and the regression coefficient was 0.122 ([**Table 1**](#Table1)).

**Table 1. Factors affecting the ripening delay (i.e. ES) achieved by viticultural practices.**

| **Antitranspirants (n = 102)**  ${ES}_{AT}=\left( \beta_{0}+\lambda_{site x vintage} \right)+\beta_{Rainfall}+\beta_{Ingredient}+\beta_{Timing}+\beta_{Second}+\varepsilon$ | | | |
| --- | --- | --- | --- |
| **Fixed effects** | **Estimate** | **SE** | ***p*-value** |
| Intercept | 0.901 | 0.451 | **0.050** |
| Slope |  |  |  |
| Rainfall | 0.122 | 0.067 | 0.078 |
| Chemical (kaolin)^1^ | -1.296 |  | **< 0.0001** |
| Timing of application^2^ |  |  |  |
| Post-fruitset | 0.284 | 0.300 | 0.347 |
| Bunch closure | -0.010 | 0.437 | 0.982 |
| Pre-veraison | 0.508 | 0.181 | **0.007** |
| Veraison | 0.164 | 0.416 | 0.695 |
| Second application | 0.901 | 0.185 | **< 0.0001** |
| **Random effects (N)** | **Variance** | **SD** |  |
| Site *x* vintage (35) | 0.207 | 0.455 |  |
|  |  |  |  |
| **Delayed pruning (n = 45)**  ${ES}_{DP}=\left( \beta_{0}+\lambda_{variety x vintage} \right)+\beta_{Pruning stage}+\beta_{{Yield}_{Control}}+\varepsilon$ | | | |
| **Fixed effects** | **Estimate** | **SE** | ***p*-value** |
| Intercept | 1.183 | 0.865 | 0.178 |
| Slope |  |  |  |
| Pruning stage^3^ |  |  |  |
| BBCH 05 (bud swelling) | 1.624 | 0.908 | 0.081 |
| BBCH 09 (budburst) | 1.543 | 0.692 | **0.033** |
| BBCH 13 (2-3 leaves) | 2.803 | 0.673 | **< 0.001** |
| BBCH 18 (7-8 leaves) | 4.265 | 0.702 | **< 0.0001** |
| Log(Yield_Control_)^4^ | -2.624 | 0.552 | **< 0.0001** |
| **Random effects (N)** | **Variance** | **SD** |  |
| Variety *x* vintage (19) | 1.258 | 1.121 |  |
|  |  |  |  |
| **Late source limitation (n = 56)**  ${ES}_{DP}=\left( \beta_{0}+\lambda_{variety} \right)+\beta_{{TSS}_{C}}+\beta_{{ES}_{Yield}}+\beta_{{Yield}_{Control}}+\varepsilon$ | | | |
| **Fixed effects** | **Estimate** | **SE** | ***p*-value** |
| Intercept | -7.490 | 2.099 | **< 0.001** |
| Slope |  |  |  |
| TSS_Control_ | 0.279 | 0.078 | **< 0.001** |
| ES_Yield_ | -0.804 | 0.125 | **< 0.0001** |
| Yield_Control_ | 0.729 | 0.111 | **< 0.0001** |
| **Random effects (N)** | **Variance** | **SD** |  |
| Variety (12) | 1.163 | 1.079 |  |

Notes: regression coefficients (β) and *p*-values are shown for each factor. Significant *p*-values (< 0.05) are highlighted in bold. Abbreviations: AT = antitranspirants; DP = delayed pruning; LSL = late source limitation; SD = standard deviation; SE = standard error.

^1^ The reference category for chemical was di-1-*p*-menthene, with the estimated coefficient set to 0.

^2^ The reference category for timing of application was ‘pre-flowering’, with the estimated coefficient set to 0.

^3^ The reference category for pruning stage was BBCH 01 (late winter dormancy), with the estimated coefficient set to 0.

^4^ Yield_Control_ was log-transformed to fit an inverse exponential relationship with the ES.

When comparing kaolin and di-1-*p*-menthene, this MA found significantly larger ripening delays as a result of di-1-*p*-menthene applications (*p* < 0.001). Estimated effects ([**Figure 4A**](#Figure4)) were 1.76 °Brix (CI: 1.33 – 2.19) for di-1-*p*-menthene and 0.46 °Brix (CI: -0.13 – 1.79) for kaolin formulates, respectively.

The ripening delay caused by ATs was also impacted by the timing of application (*p* = 0.007, [**Table 1**](#Table1)). Estimated effects of spraying at different stages are shown in [**Figure 4B**](#Figure4). Pre-veraison applications (estimated ES: 1.43 °Brix, CI: 1.03 – 1.83) led to significantly larger ES values compared to pre-flowering sprays (estimated ES: 0.92 °Brix, CI: 0.54 – 1.30), while differences were not significant for the other application stages. In addition to the timing of application, the model highlighted a significant (*p* < 0.0001) positive effect of repeating the AT application either pre-veraison or at veraison ([**Figure 4B**](#Figure4)). Average estimates for single and double applications were 0.86 °Brix (CI: 0.61 – 1.11) and 1.77 °Brix (CI: 1.43 – 2.11), respectively.

The random term (site × vintage) was significant (*p* = 0.010) and random coefficients are reported in the **Supporting Information** (Section 5.1.5, Figure 5.1.4).

### Delayed pruning

The literature search identified 64 ES values for DP distributed across 10 studies. Observations on DP were collected in Europe, Australia and New Zealand and this technique was only tested on red grape varieties (**Supporting Information**, Sections 3.1.9 to 3.1.11). Preliminary analysis of the pooled ES values ([**Figure 5A**](#Figure5)) showed that the average ripening delay caused by DP was 1.57 °Brix (CI: 1.14 – 2.00). Experimental conditions under which delayed pruning was tested were highly variable, as captured by the high degree of heterogeneity *I^2^* (92%, CI: 90% – 94%). The funnel plot ([**Figure 5B**](#Figure5)) showed some individual observations outside of the 95% confidence band. There were both large studies that reported only small effects (top-left corner of [**Figure 5B**](#Figure5)) or small studies with very large effects (bottom-right corner). However, these were random observations across multiple studies and they were in the opposite direction to the expected outcomes. Further exploration of the outliers showed that their unexpected behavior was not necessarily driven by a smaller degree of replication.

Significant factors assessed using a linear mixed model are reported in [**Table 1**](#Table1), and the residuals of the model were normally distributed ([**Figure 5C**](#Figure5)). Significant effects (*p* < 0.05) on the ES values were observed for two variables, namely pruning stage and Yield_Control_. On the contrary, there was no significant effect of TSS_Control_, vine size, pre-pruning, seasonal GDDs and precipitation. Among the random effects tested in the model, the vintage × variety interaction maximized the predictive power of the model (**Supporting Information**, Section 5.2.2) and it was significant at *p* < 0.00001. It was shown that there was almost a one-to-one relationship between variety and site in the DP dataset (**Supporting Information**, Table 5.2.1), indicating that the fitted term also accounted for site-to-site variation. The random variation on the intercept for 19 groups of vintages and varieties is shown in the **Supporting Information** (Figure 5.2.4).

The effect of pruning stage was significant at *p* < 0.0001 and had the largest contribution (η^2^ = 65.0 %) to explaining variance of primary literature (**Supporting Information**, Section 5.2.5). Model predictions ([**Figure 5D**](#Figure5)) were extrapolated for five sequential pruning stages, namely late winter dormancy, bud swelling, budbreak, 2-3 leaves unfolded and 7-8 leaves unfolded. Estimated ES values for bud swelling (0.46 °Brix, CI: -0.67 – 1.60) and budbreak (0.57 °Brix, CI: -0.30 – 1.45) were positive but the corresponding CIs included 0, as opposed to estimates for pruning at 2-3 leaves (1.85 °Brix; CI: 1.10 – 2.61) or 7-8 leaves unfolded (3.28 °Brix, CI: 2.37 – 4.19). Positive regression coefficients ([**Table 1**](#Table1)) and higher estimates ([**Figure 5D**](#Figure5)) were observed when pruning was delayed to later stages of apical bud development.

The effect of ES_Yield_ was not significant, thus this variable was excluded from the fixed effects (**Supporting Information**, Section 5.2.4). On the contrary, the regression coefficient of Yield_Control_ was significant (*p* < 0.0001, [**Table 1**](#Table1)). The best fit to describe the relationship between Yield_Control_ and ES was an inverse exponential function ([**Figure 5E**](#Figure5)).

### Late source limitation

The quantitative dataset for LSL was composed of 55 ES values collected from 10 studies, including 32 ES values in 8 studies for late defoliation and 13 ES values in 4 studies for late trimming. LSL treatments were tested in both white and red grape varieties and different countries and regions worldwide (**Supporting Information**, Sections 3.1.9 and 3.1.11). In the forest plot of LSL studies (**Figure 6A**), pooled effects were calculated both separately for late defoliation and late trimming, and for the two treatments combined. In all three cases, CIs of the pooled effects were greater than zero and the average ES values were 1.04 °Brix (CI: 0.80 – 1.29) for late defoliation and 1.51 Brix (CI: 0.57 – 2.44) for late trimming. In the funnel plot (**Figure 6B**), the distribution of the ES values against their SE suggested that there was no trace of publication bias. Levels of study heterogeneity were lower in late defoliation subgroup (*I*^2^ = 43 %) than late trimming (*I*^2^ = 79 %), and modest when the treatments were combined together (*I*^2^ = 62 %, CI: 47 – 72 %).

The model was fitted to 56 observations of late defoliation and late shoot trimming and the significant terms are reported in [**Table 1**](#Table1). The distribution of model residuals was approximately normal (**Figure 6C**), and normality was confirmed by the Shapiro-Wilk test (**Supporting Information**, Section 5.3.6). The selection procedure for random terms was identical to the datasets of ATs and DP (**Supporting Information**, Section 5.3.2) and the best random term was represented by the grape variety, the addition of which resulted in significance at *p* = 0.002. As it was the case for DP, there was a very close association between sites and varieties (**Supporting Information**, Table 5.3.1). The deviation from the model intercept for 12 grape varieties is shown in the **Supporting Information** (Figure 5.3.4).

As for the fixed effects investigated, non-significant regression coefficients ([**Table 1**](#Table1)) were observed for treatment type, timing and LAFW ratio parameters (both absolute and ES values). Environmental variables, namely GDDs, rainfall and their interaction, were also non-significant.

There was a significant effect (*p* < 0.001) of the TSS maturity at which the control and treated grapes were compared (TSS_Control_, [**Table 1**](#Table1)). The variance explained by this term (η^2^ = 20.2 %) was the second highest after yield-related variables (ES_Yield_ and Yield_Control_). Significant ripening delays (i.e. positive ES estimates with CI > 0) were associated with TSS_Control_ ≥ 23 °Brix (**Figure 6D**).

The LSL model ([**Table 1**](#Table1)) returned highly significant effects (*p* < 0.00001) for both ES_Yield_ and Yield_Control_. The regression coefficients were -0.804 for ES_Yield_ and 0.729 for Yield_Control_, indicating that ES values increased inversely to ES_Yield_ values (**Figure 6E**) and proportionally with yield conditions of control vines (**Figure 6F**). Vines with yields greater than 3 kg/m cordon were increasingly favorable to delay ripening, as shown by the CI of the fit becoming larger than 0 (**Figure 6F**). With regards to the relationship between ES values on yield and TSS (**Figure 6E**), unchanged or increased yields (i.e. ES_Yield_ ≤ 0 kg/m cordon) were positively associated with ripening delays, whereas mild yield reductions (0.5 – 1.5 kg/m cordon) led to null effects on TSS, or eventually advanced ripening when severe (> 2 kg/m cordon).

# Discussion

The aim of this research was to further evaluate the feasibility of vineyard techniques to delay ripening, needed to combat the accelerated sugar accumulation caused by changing climate conditions^3,41^. A quantitative review of the efficacy of such growing practices was lacking, and therefore MA techniques were applied to allow the analysis of data from over 40 independent experiments reported in the primary literature. MA tests the size of a treatment effect, the “effect size” ES^11^; in the present work the delaying effect (i.e., the ES) was quantified as the difference in TSS between the control grapes and grapes submitted to a treatment to delay ripening. ES values calculated from original trials of delayed ripening were collated into a global dataset and screened prior to meta-analysis following the PRISMA statement layout^20^. The application of inclusion/exclusion criteria for MA led to a database of 43 studies and 242 ES values.

The preliminary investigation of the efficiency of vineyard practices to delay ripening was undertaken using the *p*-curve and PET-PEESE methodologies, which have been proposed to test whether there is an adequate evidential value in collections of studies^25,26^. The *p*-curve technique uses the distribution of significant *p*-values as an indicator of the presence or absence of an effect^25^. The marked right-skewed *p*-curve (**Figure 2A**) built from the studies of interest suggested the presence of an adequate evidential value (i.e. a “true” delaying effect) and little interference of publication bias. The PET-PEESE procedure approximates the true ES of a treatment, calculated as the intercept of the regression between ES and SE values^26^. According to the PET-PEESE (**Figure 2C**), the ripening delay caused by the vineyard strategies investigated could be quantified as an average reduction of 1.28 °Brix at harvest. As expected, employing different types of vineyard management had a significant effect on the ripening delay (**Figure 2D**), which was investigated by the analysis of the outcomes of three individual treatments: antitranspirants, delayed pruning and late source limitation.

Linear mixed models were employed to model the ES based on variables of interest, outlined in **Section 2.6.4** and carefully selected on the basis of viticultural knowledge as well as data availability. These linear mixed models accounted for a certain portion of “random” variation^42^, which was best represented by the site and vintage. Random terms have the advantage of incorporating variation that is expected to occur in a random fashion (i.e. sites and vintages are not correlated) and too complex to summarize with a limited number of variables, and therefore to focus more directly on the effects of experimental parameters that can be controlled by growers (e.g. the timing of treatment application or treatment intensity). Non-significant coefficients of total GDDs and rainfall, fitted as fixed effects, were likely explained by significant random terms, as site and/or vintage are the main drivers of weather differences. In addition to site and vintage, grape variety was also found to be a useful random term in the DP and LSL models (**Table 1**). Because there was a close association between site and grape variety utilized in these datasets, it is reasonable to speculate that the random variation of variety partially reflected site-to-site variation.

Irrigation is a management practice commonly used in grapevine cultivation to overcome insufficient rainfall as well as manipulate canopy growth, grape yield and quality. In our study it was not possible to account for irrigation amounts due to incomplete or missing details in original papers. The influence of additional irrigation on the efficacy of vineyard practices used to delay ripening of grapes is an important research question that deserves to be investigated in future studies.

## Antitranspirants

Water loss by transpiration plays a major role in maintaining vine vitality, promoting growth and coping with environmental conditions^43^. Vine transpiration is regulated by stomata in response to root water availability through a combination of hydraulic and hormonal signals^44,45^. Gas stomatal conductance directly affects ripening, as decreases in this parameter correlate to lower assimilation of photosynthates^46^. Grape ripening is also dependent on cuticular transpiration directly occurring from grape berries, and a restriction in berry transpiration has been shown to decrease the rate of sugar accumulation^47–49^. Based on these advancements in the knowledge of water relations in ripening grapes, it has been proposed that application of ATs may reduce sugar accumulation and delay ripening^9,10^. The two prevalent active compounds utilized in AT formulations are the film-forming AT di-1-*p*-menthene, also called pinolene, and kaolin formulates, also referred to as particle film technology. Studies conducted on potted vines have provided the theoretical framework as to how ATs affect canopy performance and fruit quality^50,51^. Although experimental trials in these semi-controlled conditions depict a scenario of positive outcomes derived by the application of ATs, there is still large variation among results of in-field applications, which was the object of study by our MA. Such variation was captured by the *I^2^* when the ES values of AT trials were pooled together in the forest plot (**Figure 3A**), which indicated that average ES achieved by AT applications was positive (0.74 °Brix) and confirmed the overall efficacy of using ATs to delay ripening. Interestingly, results of meta-regression highlighted important implications regarding the choice of AT formulates as well as the timing and number of applications (**Table 1**).

Although kaolin and di-1-*p*-menthene have different modes of action, this MA allowed a better characterization of their potential effect on sugar accumulation kinetics. This MA suggested that di-1-*p*-methene formulations led to longer ripening delays (+ 1.27 °Brix) compared to kaolin (**Figure 4A**). A direct comparison of these two compounds was reported previously^52^ over three consecutive vintages, with di-1-*p*-menthene resulting in larger delays compared to kaolin in two vintages. Results obtained herein confirmed these findings and, in this case, a much larger number of observations was analyzed across different sites, varieties and vintages.

The significant effect of the timing of AT application on the ripening delay (**Table 1**) may be explained by a change in vine physiological performance arising from varying canopy size, age and management strategies^43^. Confidence intervals were higher than 0 for the estimates of all application stages, signifying positive outcomes of AT sprays to delay sugar accumulation (**Figure 4B**). However, it was shown that later applications (pre-veraison) led to significantly larger ES values compared to pre-flowering sprays. Larger ripening delays were also observed when early applications were followed by a second spray of ATs close to veraison (**Figure 4C**), whereby the second application increased the ripening delay by about 1 °Brix compared to single sprays. These results may be explained by the compensation of early effects during the subsequent vegetative growth. Lower effectiveness resulting from early applications may also be due to rainfall events, which are more frequent early in the season, since earlier studies reported that di-1-*p*-menthene remains on leaves for about 40 days^53^. In the AT model, the effects of total seasonal GDDs and rainfall were not significant and likely accounted for by the random term (site × vintage, **Table 1**). However, the timing of rainfall was not incorporated, but this dynamic variable may explain the increased efficacy of antitranspirants when re-applied close to ripening initiation.

Previous studies have demonstrated that vine yield and its relationship to leaf area (i.e. the crop load, or LAFW ratio) drive fruit ripening and define its final quality^54^. Due to missing yield data for a large unpublished study, it was not possible to investigate yield effects on the efficacy of ATs in this MA. Models for DP ([**Section 3.3.2**](#_Delayed_pruning)) and LSL ([**Section 3.3**](#_Limitation_of_carbon)**.3**) highlight the importance of considering yield conditions to fully understand the factors involved in delayed ripening using these techniques, and therefore characterization of the effect of yield levels on the use of ATs remains of great importance in future studies.

## Delayed pruning

Vineyard pruning is vital for grapevine production as it allows retention of the desired number of buds, directly affecting crop load, and control of canopy size and shape which impacts grape microclimate and facilitates vineyard mechanization^43,55^. Grapevines are traditionally pruned during winter dormancy but DP until budbreak or later has recently been regarded as another useful tool to delay ripening^9,10,56^. Accordingly, the forest plot of DP returned an average delaying effect of 1.57 °Brix, which was however derived from a highly variable dataset (**Figure 5A**).

The physiological foundation of DP (also called late pruning) is the acrotony of budbreak among buds positioned along a vertical cane, meaning that apical buds exert hormonal inhibition towards basal buds through the production and basipetal translocation of auxins^57^. DP aims to postpone pruning activities until after apical buds have burst, to exploit the inhibitory effect exerted towards basal buds and with the assumption that significant reserves would be allocated to apical buds, causing a delay in the development of basal buds destined for grape production. Original studies have investigated the effect of DP at different stages, which is a key consideration for grape growers. Nevertheless, there is little agreement on how late pruning has to be performed with respect to maintaining yield and enhancing fruit composition^10^. It is assumed that the later vines are pruned, the larger delays of vine phenology and potentially ripening are achieved, due to a greater utilization of reserves which become unavailable for the development of basal shoots for fruit production. However, previous studies have also shown overcompensation effects in vines subjected to LP^58^. In the meta-regression of DP data, regression coefficients ([**Table 2**](#Table2)) and estimates by pruning stage ([**Figure 6A**](#Figure6)) confirmed this hypothesis, with increasingly larger ripening delays achieved by pruning at later stages of development of apical buds.

Potential side-effects of DP on yield components have been discussed in earlier studies, and trends depicted by re-analyzing original studies reinforce these observations (**Supporting Information**, Figure 4.2.17). Negative consequences on yield can be explained by the fact that resources directed towards the development of apical buds would not be available for the development of fruit^58^. It was observed that the relationship between yield effects and ripening delay was not significant. On the contrary, the DP model returned a significant effect of Yield_Control_ ([**Table 1**](#Table2)), which represented the absolute yield of treated vines in original studies. Crop load levels (i.e. LAFW ratios) were also retrieved from the original studies, however these were too few to be included in the model and in their place, crop variables were selected, thus avoiding a compromise in sample size. Based on the 95 % – CI of the fit, this MA suggests that DP can be employed to delay ripening (i.e. CI > 0) when the potential yield is ≤ 2.5 kg/m cordon. Larger yields nullified the delay until about 5 kg/m cordon and eventually seemed to cause the opposite effect (i.e. advanced ripening). The increased efficacy of delayed pruning in low-yielding vines is likely to be attributed to the relationship between crop load and ripening kinetics and resembles the curvilinear relationships drawn by previous authors to describe such relationships^54^. Ripening is accelerated in low-yielding vines^59^ and faster sugar accumulation in control vines intensifies the discrepancy between TSS of control and treated grapes.

## Limitation of carbon sources during ripening

Targeted apical-to-the-cluster defoliation or shoot trimming close to veraison have been proposed as valuable tools to delay ripening^9,10^. These types of canopy management are common techniques to modify vine leaf area and are widely utilized thanks to several mechanized solutions for their ability to directly impact fruit quality^54^. Traditionally, leaf removal has been performed at flowering or at any time leading up to veraison to increase fruit exposure to sunlight, with potential benefits for grape quality parameters^15^. Shoot trimming is instead applied for practical reasons, such as controlling canopy size and shape and facilitating machinery access in the vineyard. In the past decade, several studies have explored the effect of leaf area reduction as a tool for source limitation, with the aim to reduce photosynthetic capacity and improve grape quality. Young apical leaves proximal to the shoot apex are the primarily active source of photosynthates to support sugar accumulation in ripening grapes, and the maximum photosynthetic rate is achieved when leaves are 40-days old^60–62^. It was therefore hypothesized that a reduction of apical leaf area could lead to slower sugar accumulation and thus a ripening delay^9^. The forest plot of late source limitation studies confirmed the efficacy of late source limitation to delay ripening, with an average ES of 1.16 °Brix (**Figure 6A**).

Among the variables investigated in the LSL model, yield conditions and the TSS maturity targeted for harvest significantly impacted the intensity of the ripening delay caused by LSL (**Table 1**). Importantly, the non-significant regression coefficient for treatment type indicated that late apical defoliation or late trimming can be used interchangeably without changing the ES values. The efficacy of late shoot trimming is known to be dependent on vine vigor, soil fertility an environmental factors^9^. Our meta-analysis intended to select studies as comparable as possible during the screening procedure. Further analysis, as well as additional trials on late shoot trimming, are needed in the future for a more in-depth analysis of this individual treatment. Similarly, across the explanatory variables investigated, the timing of application seemed to be of little importance in the hierarchy of factors influencing ES values.

Arguably, the difference in TSS values at which the control and ripening grapes were compared (**Figure 6D**) is explained by the shift from active sugar accumulation to passive sugar concentration via water loss occurring when TSS reaches 20 – 25 °Brix^47,48,63^. TSS differences between control and treated grapes may be exacerbated when control grapes begin to shrivel, providing a possible explanation for the proportional increase of targeted TSS and ES values. Because ripening delays may start to be evident at TSS ≥ 23 °Brix, LSL seems to be more suitable to delay ripening in late-harvested varieties, such as red grapes for medium-to-full bodied wines, rather than white varieties harvested at earlier stages.

Meta-regression highlighted the primary importance of yield conditions (i.e. absolute yield values) as well as treatment effects on yield (**Table 1**). Modelling of cross-sectional LSL observations clarified that ripening delays were achieved as a result of limiting conditions for ripening, such as when the treatment was applied to high-yielding vines, whereby the sugar accumulation process occurs at a slower pace. Vines with yields greater than 3 kg/m cordon were increasingly responsive to ripening delays, as shown by the CI of the fit becoming larger than 0 ([**Figure 6F**](#Figure8)). These findings also stress the importance of carefully planning cropping levels to avoid under-cropped situations, as these treatments are less likely to delay ripening in vines with low yield or high leaf area. It is therefore emphasized that simple tools such as achieving a suitable balance between leaf area and yield are in some cases enough to delay ripening, and should be carefully assessed before considering other techniques such as those investigated in this study^9,64^. The relationship between Yield_Control_ and ES observed in the LSL treatment displayed an opposite trend compared to DP, the latter being more effective to cause larger delays in low-yielding vines compared to high-yielding ones (**Figure 5E** versus **Figure 6F**). The different modes of action through which these techniques delay ripening possibly explain this inconsistency, since DP aims to delay grapevine phenology starting at the beginning of the season while the intent of LSL is to cause a limitation in carbon allocated to grapes at the onset of ripening or soon thereafter. With regards to the relationship between ES values on yield and TSS ([**Figure 6E**](#Figure8)), it was shown that yield reductions occurring as a consequence of vine treatments can lead to null effects when mild (0.5 – 1.5 kg/m cordon) or can advance ripening when severe (> 2 kg/m cordon). Such an impact of ES_Yield_ is a factor of tremendous importance, as the outcome of the treatment can be compromised when yield is negatively impacted by the application of late defoliation or trimming. ES_Yield_ is unpredictable and largely out of the growers’ control, whereas growers can actively adjust pruning decisions to reach yield levels favorable to delay ripening.

LAFW ratio is an important physiological indicator of sink/source relationships in the grapevine, which are linked to ripening dynamics and the final fruit composition^54,65,66^. Earlier studies have demonstrated that LAFW ratios in the range of 10 to 14 cm^2^/g are optimal to fully ripen fruit, while higher and lower ratios correspond to limiting and excessive conditions for ripening respectively^54,67^. Because the LAFW ratio is calculated as the ratio between leaf area and fruit weight (i.e. yield), these results suggest that yield conditions are the main explanatory variable for the delay of ripening obtained by LSL.

## Meta-analysis to uncover stability and drivers of agronomic practices

Our meta-analysis of techniques to delay ripening exemplifies the feasibility of using statistical methods to unlock trends across a large collection of experimental trials. Meta-analysis as a statistical method selects a group of studies characterized by the same general research question from a population following a protocol of selection criteria and aggregates them to formulate an accurate answer on the issue they have in common. With an increasing amount of data being collected and processed in agricultural research, it is expected that meta-analysis will be increasingly applied in the future. In the field of grape and wine research, several management practices other than those aiming to delay ripening have been extensively tested in field experiments. The results of these field experiments can be included into meta-analyses, enabling power of advanced statistics to be used to achieve a fuller interpretation of the factors that influence treatment efficacy and their relative importance. Further, our analysis focused on a single aspect of ripening (i.e. sugar accumulation); meta-analyses may be applied to uncover additional trends that may otherwise remain obscured such as vine physiology, yield or other quality parameters (for instance organic acids or color). Similarly, this technique may be applied to fully characterize the treatment effects used in several other crops. Trends arising by these increasingly popular meta-analytic studies should be interrogated parallel to results of physiological studies on a given management practice to provide a more comprehensive understanding of the effects of agronomic practices. The benefits of meta-analysis are even more appealing when considering the urgency of quickly identifying mitigation strategies to a fast-changing climate. Field experiments have to deal with the perennial nature of grapevines, as well as their seasonal growth and limited tested conditions (soil, variety, training system, planting density, etc.). Meta-analysis has the power to combine such unique conditions to characterize whether a treatment is effective in a rapid and accurate manner.

# Conclusions

The evidence for the need to delay ripening to reduce the pressure of climate change and its detrimental effects on grape quality has led to an increasing number of experimental trials exploring techniques to delay sugar accumulation. In addition to highly variable environmental conditions, there are often different experimental systems to which treatments are applied. This study represents the first attempt at a data-driven exploration on how these factors affect delayed ripening. In the present study, meta-analysis was applied to quantitative data collated from 43 primary studies. Delayed ripening was tackled strictly from the perspective of sugar accumulation, using the difference between TSS of control and treated grapes at harvest as a measure of ripening delays. Based on extensive research, it was assumed that slower sugar accumulation leads to benefits in terms of grape and wine quality. Relationships between effects on TSS and other quality traits of grapes may further benefit from the meta-analysis approach in future research, although different analytical methodologies utilized for the analysis of specialized metabolites in very low concentrations may represent an additional source of variation that is difficult to overcome.

A preliminary untargeted meta-analysis of 242 observations highlighted that several techniques explored in the primary literature are useful to delay sugar accumulation, decompress harvest and enhance grape quality. It was also shown the treatment efficacy varied across the different treatment categories considered, which was expected due to differences in the foundation and mechanism of action of these treatments. Further exploration of the effect of environmental and experimental conditions was carried out fitting linear mixed models on three selected treatments that would be the easiest to implement, namely antitranspirants, delayed pruning and late source limitation.

Antitranspirants represent a viable tool to delay ripening, and this meta-analysis showed that their efficacy is dependent upon the choice of active compound, with formulates of di-1-*p*-menthene considered more effective than kaolin-based sprays. As for the timing of application, spraying close to or at veraison is preferred to delay ripening, which could otherwise be obtained by a combination of early- and late applications, such as at pre-flowering and pre-veraison or at bunch closure and veraison.

Delayed ripening by pruning vines at budbreak or thereafter aims to shift grapevine phenology to a later period and was found to be dependent on the pruning stage and yield conditions. Pruning at later stages of apical bud development correlated with larger ripening delays. Additionally, it was shown that the efficacy of delayed pruning was higher in low-yielding vines compared to high-yielding ones. It was predicted that vines bearing less than 3 kg/m cordon would respond positively to delayed pruning.

Reducing leaf area either at veraison or later during ripening offers another possible option to delay ripening. There was no difference in the outcome of late defoliation and late shoot trimming, and both techniques should be considered more suitable for the production of red grapes, which are normally harvested at higher TSS. The efficacy of late source limitation treatments was dependent upon yield conditions, with larger ripening delays obtained under more limiting conditions, such as when applied to high-yielding vines. The extent to which late source limitation treatments delay ripening is also affected by treatment effects on yield, and ripening delays are achieved when yield is unchanged or increased by late defoliation or late trimming.

Intra- (i.e. vintage, site, variety) and inter-study variation is a confounding variable that is often overlooked in qualitative reviews. Techniques for meta-analysis provide a powerful tool to elucidate hitherto hidden data from within the results of multiple experimental trials, resulting in a more accurate and quantitative measure of treatment effects and uncovering aspects that can support growers’ decisions to achieve the desired quality outcome.

# Abbreviations

| **AT** | Antitranspirants |
| --- | --- |
| **CI** | Confidence intervals (95 % unless otherwise indicated) |
| **DP** | Delayed pruning |
| **ES** | Effect size (calculated as value_Control_ – value_Treated_ for all parameters) |
| **GDD** | Growing degree days |
| **LAFW** | Leaf area to fruit weight (ratio) |
| **LSL** | Late source limitation |
| **SE** | Standard error |
| **TSS** | Total soluble solids |

# References

The following are references cited in the text. A full list of references for primary studies from which data were sourced is available in the **Supporting Information**.

1. Waterhouse, A. L., Sacks, G. L. & Jeffery, D. W. *Understanding wine chemistry*. *Understanding Wine Chemistry* (John Wiley and Sons, 2016). doi:10.1002/9781118730720

2. Fraga, H., Malheiro, A. C., Moutinho-Pereira, J. & Santos, J. A. An overview of climate change impacts on European viticulture. *Food Energy Secur.* **1**, 94–110 (2012).

3. Jones, G. V., White, M. A., Cooper, O. R. & Storchmann, K. Climate change and global wine quality. *Clim. Change* **73**, 319–343 (2005).

4. Jones, G. V. & Webb, L. B. Climate change, viticulture, and wine: Challenges and opportunities. *J. Wine Res.* **21**, 103–106 (2010).

5. van Leeuwen, C. & Darriet, P. The impact of climate change on viticulture and wine quality. *J. Wine Econ.* **11**, 150–167 (2016).

6. Sadras, V. & Moran, M. A. Elevated temperature decouples anthocyanins and sugars in berries of Shiraz and Cabernet Franc. *Aust. J. Grape Wine Res.* **18**, 115–122 (2012).

7. Rienth, M. *et al.* Temperature desynchronizes sugar and organic acid metabolism in ripening grapevine fruits and remodels their transcriptome. *BMC Plant Biol.* **16**, 164 (2016).

8. Petrie, P. R., Brooke, S. J., Moran, M. A. & Sadras, V. Pruning after budburst to delay and spread grape maturity. *Aust. J. Grape Wine Res.* **23**, 378–389 (2017).

9. Palliotti, A. *et al.* Changes in vineyard establishment and canopy management urged by earlier climate-related grape ripening: A review. *Sci. Hortic. (Amsterdam).* **178**, 43–54 (2014).

10. Gutiérrez-Gamboa, G., Zheng, W. & Martínez de Toda, F. Current viticultural techniques to mitigate the effects of global warming on grape and wine quality: A comprehensive review. *Food Res. Int.* **139**, (2021).

11. Cuijpers, P. *Meta-analyses in mental health research - A practical guide*. (Pim Cuijpers Uitgeverij, 2016).

12. Wang, Y., He, L., Pan, Q. H., Duan, C.-Q. & Wang, J. Effects of basal defoliation on wine aromas: A meta-analysis. *Molecules* **23**, 1–18 (2018).

13. Lavoie-Lamoureux, A., Sacco, D., Risse, P. A. & Lovisolo, C. Factors influencing stomatal conductance in response to water availability in grapevine: A meta-analysis. *Physiol. Plant.* **159**, (2017).

14. Ilc, T., Werck-Reichhart, D. & Navrot, N. Meta-analysis of the core aroma components of grape and wine aroma. *Front. Plant Sci.* **7**, 1–15 (2016).

15. VanderWeide, J. *et al.* Impacts of pre-bloom leaf removal on wine grape production and quality parameters : A systematic review and meta-analysis. *Front. Plant Sci.* **11**, 1–13 (2021).

16. Philibert, A., Loyce, C. & Makowski, D. Assessment of the quality of meta-analysis in agronomy. *Agric. Ecosyst. Environ.* **148**, 72–82 (2012).

17. Krupnik, T. J. *et al.* Does size matter? a critical review of meta-analysis in agronomy. *Exp. Agric.* **55**, 200–229 (2019).

18. Becker, L. A. Effect size (ES). (2000). Available at: http://web.uccs.edu/lbecker/Psy590/es.htm. (Accessed: 18th February 2021)

19. McGrath, R. E. & Meyer, G. J. When effect sizes disagree: The case of *r* and *d*. *Psychol. Methods* **11**, 386–401 (2006).

20. Moher, D., Liberati, A., Tetzlaff, J., Altman, D. G. & The PRISMA Group. Preferred reporting items for systematic reviews and meta-analyses: The PRISMA statement. *PLoS Med.* **6**, e1000097 (2009).

21. Abràmoff, M. D., Magalhães, P. J. & Ram, S. J. Image processing with imageJ. *Biophotonics Int.* **11**, 36–41 (2004).

22. Passing, H. & Bablok, W. A new biochemical procedure for testing the equality of measurements from two different analytical methods. *J. Clin. Chem. Clin. Biochem.* **21**, 709–720 (1983).

23. Gladstones, J. *Viticulture and Environment*. (Winetitles, 1992).

24. Sparks, A. H., Hengl, T. & Nelson, A. GSODR: Global summary daily weather data in R. *J. Open Source Softw.* **2**, 177 (2017).

25. Simonsohn, U., Nelson, L. D. & Simmons, J. P. P-curve: A key to the file-drawer. *J. Exp. Psychol. Gen.* **143**, 534–547 (2014).

26. Stanley, T. D. & Doucouliagos, H. Meta-regression approximations to reduce publication selection bias. *Res. Synth. Methods* **5**, 60–78 (2014).

27. Stanley, T. D. Limitations of PET-PEESE and other meta-analysis methods. *Soc. Psychol. Personal. Sci.* **8**, 581–591 (2017).

28. Sidik, K. & Jonkman, J. N. A comparison of heterogeneity variance estimators in combining results of studies. *Stat. Med.* **26**, 1964–1981 (2007).

29. Lorenz, D. H. *et al.* Growth stages of the grapevine: Phenological growth stages of the grapevine (*Vitis vinifera* L. ssp. vinifera) - Codes and descriptions according to the extended BBCH scale. *Aust. J. Grape Wine Res.* **1**, 100–103 (1995).

30. Keller, M. & Shrestha, P. M. Solute accumulation differs in the vacuoles and apoplast of ripening grape berries. *Planta* **239**, 633–642 (2014).

31. Parker, A. K., Hofmann, R. W., van Leeuwen, C., Mclachlan, A. R. G. & Trought, M. C. T. Leaf area to fruit mass ratio determines the time of veraison in Sauvignon Blanc and Pinot Noir grapevines. *Aust. J. Grape Wine Res.* **20**, 422–431 (2014).

32. Wickham, H. *ggplot2: Elegant graphics for data analysis*. (Springer International Publishing, 2016).

33. Graul, C. leafletR: Interactive web-maps based on the Leaflet JavaScript Library. (2016). Available at: https://rdrr.io/cran/leafletR/. (Accessed: 19th February 2021)

34. Sievert, C. *Interactive web-based data visualization with R, plotly and shiny*. (Chapman and Hall/CRC, 2020).

35. Balduzzi, S., Rücker, G. & Schwarzer, G. How to perform a meta-analysis with R: A practical tutorial. *Evid. Based. Ment. Health* **22**, 153–160 (2019).

36. Viechtbauer, W. Conducting meta-analyses in R with the metafor. *J. Stat. Softw.* **36**, 1–48 (2010).

37. Kuznetsova, A., Brockhoff, P. B. & Christensen, R. H. B. lmerTest package: Tests in linear mixed effects models. *J. Stat. Softw.* **82**, 1–26 (2017).

38. Sarkar, D. *Lattice: multivariate data visualization with R*. (Springer-Verlak, 2008). doi:10.1007/978-0-387-75969-2

39. Bates, D., Mächler, M., Bolker, B. M. & Walker, S. C. Fitting linear mixed-effects models using lme4. *J. Stat. Softw.* **67**, (2015).

40. Lüdecke, D. ggeffects: Tidy data frames of marginal rffects from regression models. *J. Open Source Softw.* **3**, 772 (2018).

41. Webb, L. B., Whetton, P. H. & Barlow, E. W. R. Modelled impact of future climate change on the phenology of winegrapes in Australia. *Aust. J. Grape Wine Res.* **13**, 165–175 (2007).

42. Gbur, E. E. *et al.* *Analysis of Generalized Linear Mixed Models in the Agricultural and Natural Resources Sciences*. (2012).

43. Keller, M. *The science of grapevines*. (Academic Press, 2020). doi:https://doi.org/10.1016/C2013-0-06797-7

44. Gambetta, G. A. *et al.* The physiology of drought stress in grapevine: towards an integrative definition of drought tolerance. *J. Exp. Bot.* **71**, 4658–4676 (2020).

45. Zhang, L., Marguerit, E., Rossdeutsch, L., Ollat, N. & Gambetta, G. A. The influence of grapevine rootstocks on scion growth and drought resistance. *Theor. Exp. Plant Physiol.* **28**, 143–157 (2016).

46. Flexas, J., Escalona, J. M. & Medrano, H. Down-regulation of photosynthesis by drought under field conditions in grapevine leaves. *Aust. J. Plant Physiol.* **25**, 893–900 (1998).

47. Keller, M., Zhang, Y., Shrestha, P. M., Biondi, M. & Bondada, B. R. Sugar demand of ripening grape berries leads to recycling of surplus phloem water via the xylem. *Plant, Cell Environ.* **38**, 1048–1059 (2015).

48. Zhang, Y. & Keller, M. Discharge of surplus phloem water may be required for normal grape ripening. *J. Exp. Bot.* **68**, 585–595 (2017).

49. Tilbrook, J. & Tyerman, S. D. Hydraulic connection of grape berries to the vine: Varietal differences in water conductance into and out of berries, and potential for backflow. *Funct. Plant Biol.* **36**, 541–550 (2009).

50. Frioni, T. *et al.* Understanding kaolin effects on grapevine leaf and whole-canopy physiology during water stress and re-watering. *J. Plant Physiol.* **242**, 1–12 (2019).

51. Fahey, D. J. & Rogiers, S. Y. Di-1-*p*-menthene reduces grape leaf and bunch transpiration. *Aust. J. Grape Wine Res.* **25**, 134–141 (2018).

52. Brillante, L. *et al.* Comparing kaolin and pinolene to improve sustainable grapevine production during drought. *PLoS One* **11**, 1–19 (2016).

53. Palliotti, A., Poni, S., Berrios, J. G. & Bernizzoni, F. Vine performance and grape composition as affected by early-season source limitation induced with anti-transpirants in two red *Vitis vinifera* L. cultivars. *Aust. J. Grape Wine Res.* **16**, 426–433 (2010).

54. Kliewer, W. M. & Dokoozlian, N. K. Leaf area/crop weight ratios of grapevines: Influence on fruit composition and wine quality. *Am. J. Enol. Vitic.* **56**, 170–181 (2005).

55. Iland, P. G., Dry, P., Proffitt, T. & Tyerman, S. D. *The grapevine: from the science to the practice of growing vines for wine*. (Patrick Iland Wine Promotions, 2011).

56. Novello, V. & de Palma, L. Viticultural strategies to reduce alcohol levels in wine. in *Alcohol level reduction in wine - Oenoviti International Network* (Vigne et Vin Publications Internationales, 2013).

57. Bangerth, F. Dominance among fruits/sinks and the search for a correlative signal. *Physiol. Plant.* **76**, 608–614 (1989).

58. Gatti, M. *et al.* Phenology, canopy aging and seasonal carbon balance as related to delayed winter pruning of *Vitis vinifera* L. cv. Sangiovese grapevines. *Front. Plant Sci.* **7**, 659 (2016).

59. Cameron, W. *et al.* Is advancement of grapevine maturity explained by an increase in the rate of ripening or advancement of veraison? *Aust. J. Grape Wine Res.* **27**, 1–14 (2021).

60. Candolfi-Vasconcelos, M. C. & Koblet, W. Yield, fruit quality, bud fertility and starch reserves of the wood as a function of leaf removal - evidence of compensation and stress recovering. *Vitis* **29**, 199–221 (1990).

61. Hunter, J., Skrivan, R. & Ruffner, H. P. Diurnal and seasonal physiological changes in leaves of *Vitis vinifera* L.: CO_2_ assimilation rates, sugar levels and sucrolytic enzyme activity. *Vitis* **33**, 189–195 (1994).

62. Poni, S. & Intrieri, C. Grapevine photosynthesis: Effects linked to light radiation and leaf age. *Adv. Hortic. Sci.* **15**, 5–15 (2001).

63. Tilbrook, J. & Tyerman, S. D. Cell death in grape berries: Varietal differences linked to xylem pressure and berry weight loss. *Funct. Plant Biol.* **35**, 173–184 (2008).

64. Poni, S. *et al.* Grapevine quality: A multiple choice issue. *Sci. Hortic. (Amsterdam).* **234**, 445–462 (2018).

65. Petrie, P. R., Trought, M. C. T. & Howell, G. S. Fruit composition and ripening of Pinot Noir (*Vitis vinifera* L.) in relation to leaf area. *Aust. J. Grape Wine Res.* **6**, 46–51 (2000).

66. Parker, A. K., Hofmann, R. W., van Leeuwen, C., McLachlan, A. R. G. & Trought, M. C. T. Manipulating the leaf area to fruit mass ratio alters the synchrony of total soluble solids accumulation and titratable acidity of grape berries. *Aust. J. Grape Wine Res.* **21**, 266–276 (2015).

67. Bravdo, B., Hepner, Y., Loinger, C., Cohen, S. & Tabacman, H. Effect of irrigation and crop level on growth, yield and wine quality of Cabernet Sauvignon. *Am. J. Enol. Vitic.* **36**, 132–139 (1985).

# Conflict of Interest

*The authors declare that the research was conducted in the absence of any commercial or financial relationships that could be construed as a potential conflict of interest*.

# Data availability statement

The databases of primary observations are available online in Figshare at the following link: <https://figshare.com/s/c00dab37bdb8e3a0e2df>. Each database comprises a sheet with a list and description of variables.

# Author Contributions

PP, FG, ND, KW and CF conceived the study; PP and FG collated data from primary studies; PP and FG analyzed the data and RS helped with linear mixed models; all authors were involved in data interpretation; PP drafted the manuscript; all authors have contributed to writing the manuscript and have read and accepted its final version.

# Acknowledgments

The authors gratefully acknowledge all the researchers who shared raw data, increasing the accuracy of our meta-analysis, Darren Fahey for providing unpublished data, Luigi Mariani for his precious contribution to retrieve weather data for some missing studies, Gregory Jones and German Puga for helpful discussion about weather and climate indices, and Leigh Schmidtke who revised the content of the paper prior to submission.

This research was conducted by the Australian Research Council Training Centre for Innovative Wine Production ([www.arcwinecentre.org.au](http://www.arcwinecentre.org.au); project number IC170100008), funded by the Australian Government with additional support from Wine Australia ([www.wineaustralia.com](http://www.wineaustralia.com/); project number PPA002459), Waite Research Institute, E. & J. Gallo Winery, and industry partners. The University of Adelaide is a member of the Wine Innovation Cluster.

# Supplementary data

Supporting information is available in a HTML document at this Figshare link (**the file needs to be downloaded first and then opened in any internet browser**):

<https://figshare.com/s/c00dab37bdb8e3a0e2df>

The document reports exploratory analysis and meta-regression for antitranspirants, delayed pruning and late source limitation, and includes the R code used for linear mixed models.
